# Intention to reduce dietary salt and its influencing factors in middle-aged and older hypertensive patients: a theory of planned behavior-based cross-sectional study

**DOI:** 10.3389/fpubh.2026.1765900

**Published:** 2026-03-03

**Authors:** Yaqi Wen, Xia Li, Ya Shi, Lei Luo, Du Zhang, Zumin Shi, Manoj Sharma, Yong Zhao

**Affiliations:** 1 School of Public Health, Chongqing Medical University, Chongqing, China; 2 Ministry of Education Key Laboratory of Child Development and Disorders, Chongqing Key Laboratory of Child Rare Diseases in Infection and Immunity, Department of Clinical Nutrition, Children's Hospital of Chongqing Medical University, National Clinical Research Center for Child Health and Disorders, Chongqing, China; 3 Research Center for Medicine and Social Development, Chongqing Medical University, Chongqing, China; 4 Research Center for Public Health Security, Chongqing Medical University, Chongqing, China; 5 Nutrition Innovation Platform-Sichuan and Chongqing, Chongqing, China; 6 Department of Human Nutrition, College of Health Science, Qatar University, Doha, Qatar; 7 Department of Social and Behavioral Health, School of Public Health, University of Nevada, Las Vegas, NV, United States; 8 Department of Internal Medicine, Kirk Kerkorian School of Medicine, University of Nevada, Las Vegas, NV, United States; 9 Chongqing Key Laboratory of Child Nutrition and Health, Children’s Hospital of Chongqing Medical University, Chongqing, China

**Keywords:** dietary salt reduction intention, hypertension, influencing factors, middle-aged and older adults, structural equation modeling, theory of planned behavior

## Abstract

**Background:**

Excessive dietary salt intake is a key controllable risk factor for hypertension. Despite the clear clinical benefits of reducing salt intake, overcoming the “knowledge-action” gap among patients with hypertension poses a significant public health challenge. The Theory of Planned Behavior (TPB) is a powerful framework for understanding the psychological factors that influence behavioral intentions. However, studies based on TPB that explore dietary intentions to reduce salt intake in middle-aged and older hypertension patients have not been well described nor well studied in previous literature.

**Objective:**

Using the Theory of Planned Behavior (TPB) as the theoretical framework and Structural Equation Modeling (SEM) as the methodological basis, we aimed to examine the status of salt reduction dietary intention and its influencing factors in middle-aged and older hypertensive patients.

**Methods:**

From March to November 2023, a total of 558 middle-aged and older hypertensive patients across 38 districts and counties in Chongqing Municipality, China, were interviewed using a face-to-face questionnaire with a Likert scale. SEM was used to explore the relationship between the salt reduction intention (INT) of middle-aged and older hypertensive patients aged 45 years and above and their attitude toward salt reduction (ATT), subjective norms of salt reduction (SN), and perceived behavioral control of salt reduction (PBC), as well as the bidirectional correlation between ATT, SN, and PBC.

**Results:**

Among the 558 participants, 55.2% were female, 61.3% were older, and 38.7% were middle-aged; 44.1% were the primary home cook, and 48% had a junior high school education or above. Overall 70.8% reported an intention to reduce salt in their diets. Attitudes toward a salt-reducing diet (*β* = 0.22, *p <* 0.05) and perceived behavioral control (*β* = 0.70, *p <* 0.05) positively affected salt-reducing diet intentions, with perceived behavioral control showing the strongest effect. Subjective norm (*β* = 0.14, *p* > 0.05) did not significantly affect intentions to reduce dietary salt.

**Conclusion:**

This study highlights the central influence of salt-reducing dietary attitudes and perceived behavioral control in salt-reducing dietary intentions among middle-aged and older hypertensive patients. Future nutritional health education should prioritize strategies to strengthen intention and perceived behavioral control.

## 1 Introduction

Hypertension, one of the most common chronic non-communicable diseases, is a major risk factor for cardiovascular disease mortality worldwide (1). According to statistics, among approximately 1.4 billion hypertensive patients worldwide, the blood pressure control rate is only 14%, and about 80% of patients do not receive adequate treatment (2). The burden of hypertension in China is particularly heavy, with about 256.7 million adults aged 30–79 years, an age-standardized prevalence rate of 27%, and a knowledge and standardized treatment rate of only 39 and 16%, respectively (3). The situation is even more serious in some regions, such as Chongqing, where the prevalence of hypertension among community residents aged 30–79 years is as high as 35.07%, and the control rate is only 14.6% (4), posing a major public health challenge.

High dietary sodium intake is a modifiable risk factor for hypertension. The global average salt intake of adults (approximately 10.78 g/day) far exceeds the upper limit of 5 g/day recommended by the WHO (5), and the salt intake of Chinese residents is nearly twice the recommended amount (6). Although residents’ attitudes toward salt reduction generally recognize the need for salt reduction, knowledge limitations and low rates of behavioral change reveal a persistent disconnect between “knowledge-attitude-practice (KAP)” (7). This gap highlights the limitations of interventions focusing solely on knowledge dissemination, pointing to the necessity of exploring the underlying psychosocial determinants of behavior.

Most existing studies have focused on KAP in relation to salt reduction and hypertension, paying particular attention to individual knowledge and attitudes. However, key factors such as behavioral control and the environment are often ignored (8–10). Explaining and bridging the “knowledge-action” gap is a central challenge in public health. TPB is a powerful theoretical framework that can be used to explain and predict behavioral intentions through the three core concepts: behavioral attitudes, subjective norms, and perceived behavioral control. It has also demonstrated excellent explanatory power and predictive potential in the field of health behavior (11).

However, little research has applied the TPB to understand the intention to reduce dietary salt intake among middle-aged and older hypertensive patients. To address this gap, this study aimed to develop and validate a TPB-based model of salt-reduction intention in hypertensive patients aged 45 years and above in Chongqing, China. Using SEM, we sought to elucidate the pathways and effect sizes of attitude toward salt reduction, subjective norms, and perceived behavioral control on behavioral intention. The findings are expected to reveal the formation mechanism of salt-reduction intention and provide a scientific basis for developing more targeted and effective behavioral interventions for hypertension management.

**Figure 1:**
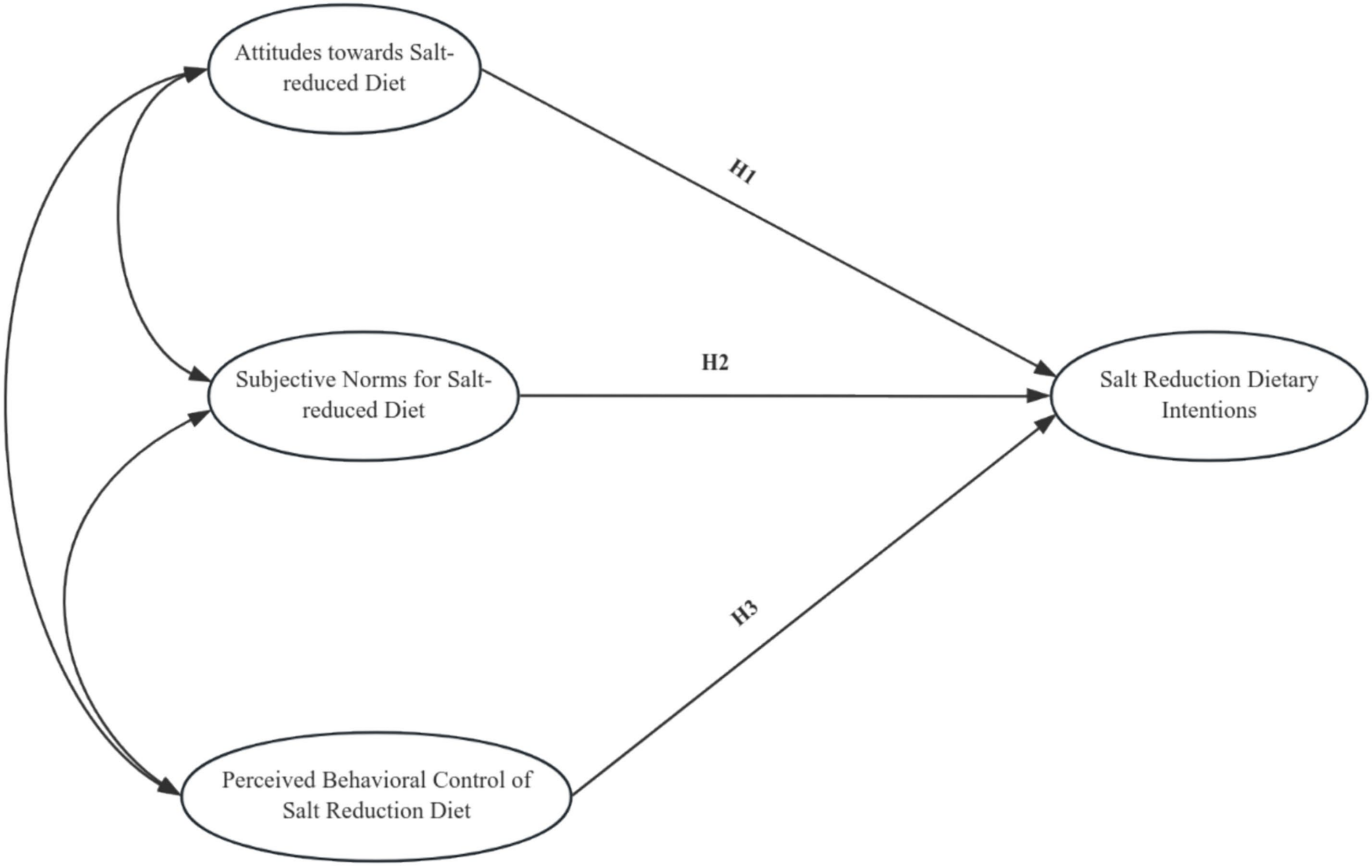
Model hypothesis testing.

We propose the following hypotheses based on the theoretical framework of TPB and the aim of this research (Figure 1).

*Hypothesis 1 (H1)*: The more robust the attitude about a salt-reduction diet, the greater the intention to reduce salt intake among middle-aged and older hypertensive patients.

*Hypothesis 2 (H2)*: The more robust the subjective norms regarding salt-reduction diet, the greater the intention to reduce salt intake among middle-aged and older hypertensive patients.

*Hypothesis 3 (H3)*: The more robust the perceived behavioral control regarding salt reduction diet, the greater the intention will be to reduce salt intake among middle-aged and older hypertensive patients.

In addition, we studied the interrelationship between salt-reducing diet attitude, salt-reducing diet competent norms, and perceived behavioral control of salt-reducing diet.

## 2 Methods

### 2.1 Study design and participants

This cross-sectional study was conducted from March 2023 to November 2023. Convenience sampling was employed to select a study population of middle-aged and older hypertensive patients (aged 45 years or older) from 38 districts and counties in Chongqing Municipality (*n =* 558). Data was collected via a face-to-face questionnaire developed for this study.

### 2.2 Eligibility criteria

Participants were included if they met the following criteria: aged 45 years or older; a prior diagnosis of hypertension at a township health center, community health service center, or equivalent facility at the monitoring site; and a mean systolic blood pressure ≥140 mmHg (1 mmHg = 0.133 kPa), a mean diastolic blood pressure ≥90 mmHg, or self-reported use of antihypertensive medication within the past 2 weeks. All participants were required to voluntarily consent to the survey. Individuals with mental disorders or significant difficulty communicating were excluded.

### 2.3 Measures

A self-administered questionnaire, based on the Theory of Planned Behavior (TPB) framework, was developed for this study. It comprised two parts: (a) essential demographic characteristics of the hypertensive patients (e.g., gender, ethnicity, place of residence, education level, and family history of hypertension) and (b) scales measuring TPB constructs. Scale development involved an extensive review of domestic and international literature on salt reduction intentions and behaviors. An initial item pool was established, refined through research team discussion and expert validation, and then pilot-tested. Data from 389 valid questionnaires in the pre-survey were analyzed using SPSS 27.0 and Amos 28.0 software. The entire scale showed excellent internal consistency (Cronbach’s alpha = 0.9000). Confirmatory Factor Analysis (CFA) indicated an acceptable model fit: *χ*^2^/df = 3.410, RMR = 0.056, RMSEA = 0.079, and GFI = 0.924 (12). Based on the statistical analysis results and expert judgement, the first draft was refined to form the final scale “Intention to Reduce Salt Diet Scale for Middle-aged and Older Hypertensive Patients in Chongqing Municipality Aged 45 Years and Above.”

The TPB scale consisted of four subscales with a total of 13 items: intention (3 items), perceived behavioral control over (3 items), subjective norms (3 items), and attitude (4 items). All items were rated on a Likert-type scale, with higher scores indicating stronger agreement with the statement.

### 2.4 Quality control

During the survey design stage, the theoretical intention model was constructed, and subscales were designed for each of the model’s four core variables using the principles and methods of scale development. Reliability and validity tests were carried out, and any subscales with poor measurement performance were revised and improved. Before formally carrying out the cross-sectional surveys, the survey researchers underwent 1 day of unified and rigorous centralized professional training. Verification and proofreading were always conducted during the survey implementation stage and the data collation and analysis stage. Any mistakes, omissions, or logical errors found were revised promptly, and the questionnaire entries were double-checked. A total of 590 questionnaires were collected from middle-aged and older hypertensive patients. Of these, 12 refused to participate in the survey, and 20 questionnaires were excluded due to many missing values. The final number of valid questionnaires collected was 558.

### 2.5 Statistical analysis

The data were cleaned and analyzed using Stata 18.0 and Amos 28.0. Continuous variables are presented as the mean ± standard deviation, and categorical variables as the frequency (percentage). For descriptive purposes, participants were categorized into “high-intention” and “low-intention” groups based on the median score of the salt-reduction intention scale, to profile their distribution. Group differences in demographic characteristics were examined using an independent samples *t*-test or one-way ANOVA, with *post-hoc* tests applied where appropriate. Separate multiple linear regression models were developed for each of the four variables (attitudes, subjective norms, perceived behavioral control, and intentions) based on the results of the univariate analyses (*p <* 0.05), to identify demographic factors that were independent predictors of each construct. SEM was employed to test the hypothesized relationships based on the TPB. In the model, salt-reduction intention (as a continuous variable) was regressed onto three constructs: salt-reducing dietary attitudes, subjective norms, and perceived behavioral control. Bivariate correlations among these TPB constructs were also assessed. Model fit was evaluated using standard indices (e.g., *χ*^2^/df, CFI, TLI, and RMSEA). All statistical tests were two-tailed, and *p <* 0.05 was considered significant.

## 3 Results

### 3.1 Participants’ characteristics

A total of 590 middle-aged and older patients with hypertension completed the questionnaire. Twelve patients refused to participate in the survey, resulting in a completion rate of 98%. Twenty questionnaires were excluded due to a high number of missing values, leaving 558 valid responses and a validity rate of 96.5%.

Among the participants, 55.2% were female, 48% had completed junior high school or higher, and 73.5% were married (Table 1). Nearly half (44.1%) were the primary food preparers in their households. A total of 50.5% reported no chronic comorbidities other than hypertension. The majority of the participants (61.3%) were older adults (≥65 years).

**Table 1:** Basic characteristics of survey respondents (*n =* 558).

Factors	Groups	*n*	*n* (%)
Salt reduction dietary intentions	No	163	29.2
	Yes	395	70.8
Sex	Women	308	55.2
	Man	250	44.8
Ethnicity	Ethnic Han	512	91.8
	Nation minority	46	8.2
Education	Primary and below	290	52.0
	Junior	151	27.1
	High school/secondary/vocational	79	14.2
	College/university above	38	6.8
Marital status	Unmarried	3	0.5
	Married	410	73.5
	Divorcee	38	6.8
	widowhood	107	19.2
Home cooks	Oneself	246	44.1
	Mate	139	24.9
	Oneself or mate	41	7.3
	Woman and son-in-law	108	19.4
	others	8	1.4
	Babysitter or carer	16	2.9
Urban-rural clusters	CC	164	29.4
	MCNA	154	27.6
	NCTGRTC	165	29.6
	SCWMTC	75	13.4
Suffering from other chronic diseases	No	282	50.5
	Yes	276	49.5
Age grouping	Middle-aged adults	216	38.7
	Older adults	342	61.3
Use of low-sodium salt	Never	211	37.8
	Rarely	148	26.5
	Sometimes	106	19
	Often	93	16.7
Use of a measured salt spoon	Never	365	65.4
	Rarely	90	16.1
	Sometimes	63	11.3
	Often	40	7.2
Checking sodium content on food labels	Never	304	54.5
	Rarely	119	21.3
	Sometimes	87	15.6
	Often	48	8.6

CC, Central City; MCNA, Main City New Area; NCTGRTC, Northeast Chongqing Three Gorges Reservoir Town Cluster; SCWMTC, Southeast Chongqing Wuling Mountains Town Cluster.

### 3.2 Univariate analysis

An independent sample *t*-test showed statistically significant differences in salt reduction dietary attitudes, subjective norms, perceived behavioral control, and intention scores between ethnic groups (*p <* 0.05). There were also statistically significant differences in perceived behavioral control scores for salt reduction between genders, with males scoring slightly lower than females in this domain (*p <* 0.05). One-way ANOVA revealed statistically significant differences across the urban–rural clusters in scores for all four TPB constructs: salt-reduction dietary attitude, perceived behavioral control, subjective norms, and intention (all *p <* 0.05). Additionally, statistically significant differences were observed in salt reduction dietary attitudes and intention among groups with different family histories of hypertension and occupational statuses (*p <* 0.05). When grouped by education level and marital status, differences in subjective norms scores for salt reduction were found (*p <* 0.05). Furthermore, statistically significant differences in perceived behavioral control scores for salt reduction and attitudes toward salt reduction were observed among those who were primary home cooks (*p <* 0.05, see Table 2).

**Table 2:** Univariate associations between demographics and TPB constructs (*n =* 558).

Variables	PBC		SN		INT		ATT	
	*t/F*	*P*	*t/F*	*P*	*t/F*	*P*	*t/F*	*P*
Gender	2.564	**0.011**	0.065	0.948	1.024	0.306	0.120	0.904
Age grouping	0.032	0.974	−1.755	0.080	0.422	0.673	−1.424	0.155
Family History of Hypertension	2.896	0.056	2.801	0.062	3.192	**0.042**	3.223	**0.041**
Education	0.338	0.798	2.865	**0.036**	1.861	0.135	1.323	0.266
Income	0.153	0.961	2.306	0.057	0.156	0.960	1.642	0.162
Occupation	0.933	0.471	2.571	0.018	3.117	**0.005**	3.196	**0.004**
Suffering from other chronic diseases	−0.278	0.781	−0.823	0.411	−0.690	0.491	−1.042	0.298
Ethnicity	3.203	**0.001**	2.532	**0.012**	3.046	**0.002**	3.648	**0.000**
Marital status	0.241	0.868	4.186	0.006	0.316	0.814	2.433	0.064
Urban–rural clusters	5.142	**0.002**	4.020	**0.006**	10.590	**0.000**	14.827	**0.000**
Home cooks	2.513	**0.029**	1.516	0.183	0.819	0.536	3.077	**0.009**

Bold *p*-values indicate statistical significance at *p <* 0.05.

### 3.3 Multiple linear regression

Multiple linear regression analysis revealed that demographic characteristics, such as residence (urban–rural) and family history of hypertension, were the primary influencing factors of salt-reduction diet intention (*p <* 0.05). Gender and residence were associated with perceived behavioral control of salt reduction (*p <* 0.05). Residence, marital status, and educational attainment were correlated with subjective norms of salt reduction (*p <* 0.05). Furthermore, residence and family history of hypertension were related to the attitudes toward salt reduction (*p <* 0.05) (Table 3).

**Table 3:** Multiple linear regression analysis of different demographic characteristics (*n =* 558).

Implicit variable	Independent variable	Regression coefficient		*t*	*P*
		*B*	*β*		
PBC	Gender	−0.143	−0.101	−2.393	**0.017**
	Urban–rural clusters	−0.104	−0.152	−3.641	**0.000**
SN	Urban–rural clusters	−0.070	−0.115	−2.735	**0.006**
	Marital status	−0.094	−0.121	−2.897	**0.004**
	Education	0.062	0.093	2.210	**0.028**
INT	Urban–rural clusters	−0.148	−0.225	−5.468	**0.000**
	Family History of Hypertension	0.119	0.101	2.442	**0.015**
ATT	Urban–rural clusters	−0.169	−0.263	−6.440	**0.000**
	Family History of Hypertension	0.094	0.081	1.980	**0.048**

Bold *P*-values indicate statistical significance at *P <* 0.05.

### 3.4 Validation and factor analysis

#### 3.4.1 Convergent validity

In the salt-reduced diet model, the mean–variance extraction AVE values of the three factors of salt-reduced diet attitude, salt-reduced diet perceived behavioral control, and salt-reduced diet intention were all greater than 0.5. The subjective norms of the salt-reduced diet were also very close to the statistical cut-off value of 0.5. The combined CR values of the reliabilities are greater than 0.7, indicating that the scale data in the present analysis have good convergent validity (Table 4).

**Table 4:** Convergent validity of the questionnaire model (*n =* 558).

Dimension	Entry	Estimate	AVE	CR
ATT	ATT1	0.874	0.649	0.880
	ATT2	0.872		
	ATT3	0.753		
	ATT4	0.710		
PBC	PBC3	0.736	0.548	0.782
	PBC2	0.635		
	PBC1	0.836		
INT	INT2	0.856	0.536	0.767
	INT3	0.798		
	INT1	0.487		
SN	SN1	0.720	0.480	0.734
	SN2	0.709		
	SN3	0.647		

#### 3.4.2 Structural validity

The model’s overall fit was evaluated using the following standard fitting indices. The results were as follow: RMSEA = 0.083, AGFI = 0.886, GFI = 0.926, *χ*^2^/df = 4.822. These values collectively indicate an acceptable overall model fit (see Table 5).

**Table 5:** Values of each fitness indicator for the questionnaire model (construct validity; *n =* 558).

Statistical test	Criteria for adaptation or threshold value	Model test results	Model fit judgement
Absolute fit indices			
RMR	<0.05	0.032	Yes
RMSEA	<0.08 favorable, < 0.1 acceptable	0.083	Yes
GFI	>0.9 desirable, > 0.8 acceptable	0.926	Yes
*χ*^2^/df	<2 favorable, < 3 ordinary, < 5 acceptable	4.822	Yes
AGFI	>0.9 desirable, > 0.8 acceptable	0.886	Yes
Incremental fit index			
NFI	>0.9	0.926	Yes
RFI	>0.9	0.902	Yes
IFI	>0.9	0.940	Yes
TLI (Tucker-Lewis Index)	>0.9	0.920	Yes
CFI	>0.9	0.940	Yes
Simple fit statistics			
PGFI	>0.5	0.600	Yes
PNFI	>0.5	0.700	Yes
PCFI	>0.5	0.711	Yes

#### 3.4.3 Discriminant validity

Discriminant validity was assessed by comparing the square root of the AVE for each factor with the correlations between that factor and all others. For the attitude factor, the square root of the AVE (0.806), exceeded its highest correlation with any other factor (max |r| = 0.030). Similarly, for the subjective norms factor, the square root of the AVE (0.693) was greater than its highest correlation with other factors (max|r| = 0.027). These findings confirm that the scale possesses good discriminant validity (Table 6).

**Table 6:** Discriminant validity (*n =* 558).

Items	ATT	SN	PBC
ATT	0.649		
SN	0.028^***^	0.480	
PBC	0.030^***^	0.027^***^	0.548
AVE square root	0.806	0.693	0.740

***p < 0.01.

### 3.5 Structural equation modeling results

**Figure 2:**
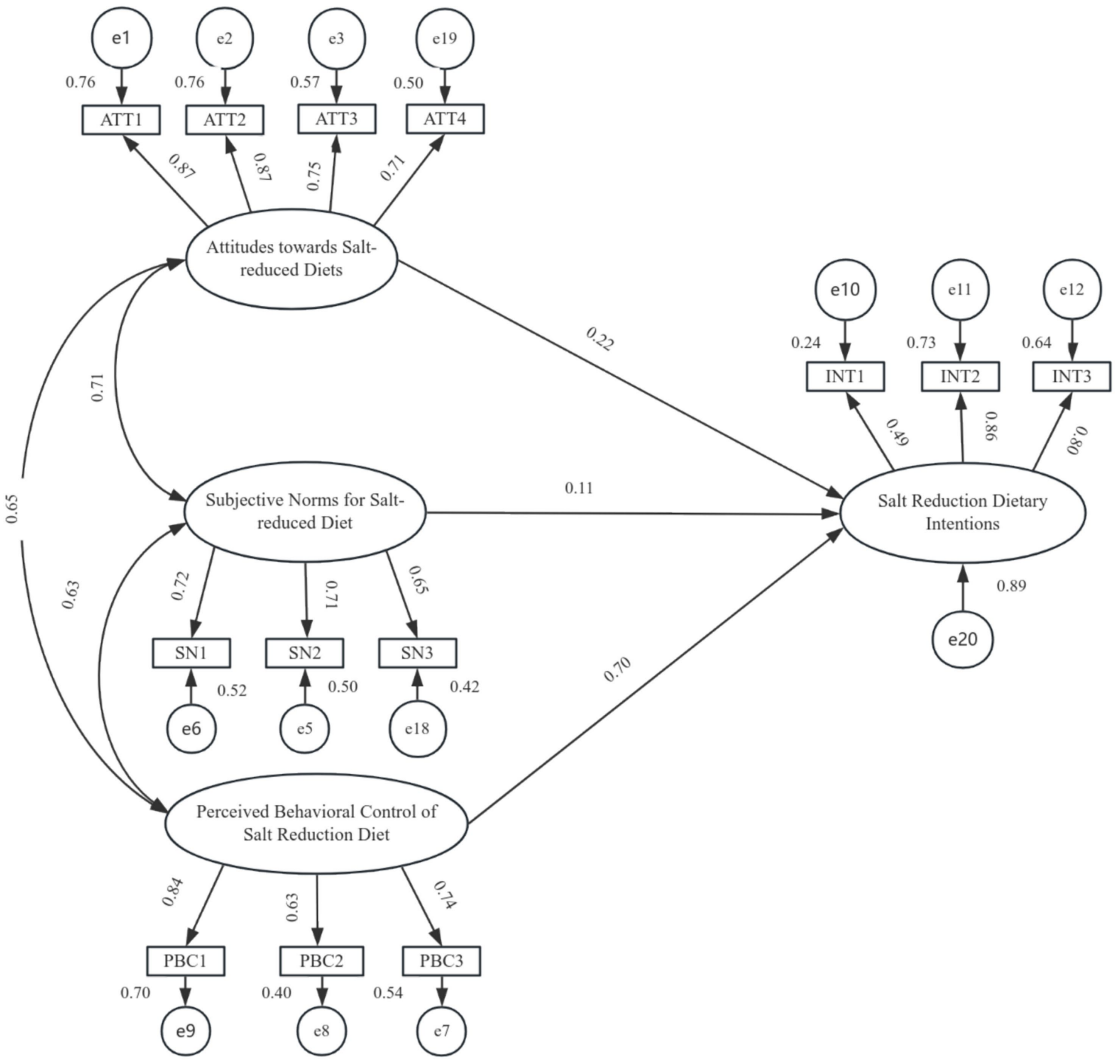
Structural equation modeling of factors influencing salt reduction dietary intentions in older hypertensive patients. Rectangles represent measured variables; ovals represent latent variables; circles represent residual terms; values on arrows for individual items represent standardized coefficients. All paths were significant (*p <* 0.05). PBC, Perceived Behavioral Control of Salt-Reduced Diet; SN, Subjective Norms of Salt-Reduced Diet; INT, Intention to Reduce Salt-Reduced Diet; ATT, Attitude Toward Salt-Reduced Diet.

The final structural model is presented in Figure 2. Path coefficient tests for the effects of attitude, subjective norms, and perceived behavioral control on intention are detailed in Table 7. The analysis revealed significant direct effects of attitude and perceived behavioral control on intention. Specifically:

- H1 was supported: Attitudes toward a salt-reduction diet had a significant positive direct effect on intention (*β* = 0.22, *p* < 0.001).
- H3 was supported: Perceived behavioral control over salt reduction had a significant positive direct effect on intention (*β* = 0.70, *p* < 0.001).

**Table 7:** Table of estimated parameters of SEM model for salt reduction diet intention and its influencing factors (*n =* 558).

Latent variable		Unstd.	S. E.	T C. R	*P*	Std.	Result
Salt Reduction Dietary Intentions	← Attitudes toward salt-reduced diets	0.220	0.055	3.962	***	0.222	Acceptance
	← Subjective norms for salt-reduced diets	0.140	0.074	1.892	0.058	0.114	Rejection
	← Perceived Behavioral Control of Salt Reduction Diet	0.744	0.066	11.223	***	0.698	Acceptance

Unstd., unstandardized; S. E., standard error; Std., standardized. ***p < 0.01.

This indicates that a one-unit increase in the standardized score for attitudes is associated with a 0.22-unit increase in the intentions score, while a one-unit increase in perceived behavioral control is associated with a 0.70-unit increase in the intentions.

## 4 Discussion

This study investigated the psychosocial factors influencing the intention to reduce dietary salt in hypertensive patients aged 45 and over in Chongqing Municipality. The result showed a significant association between the psychosocial factors influencing salt reduction intention, consistent with previous domestic and international studies (13, 14). Based on the TPB, the study revealed that salt reduction intentions were associated with attitude toward salt reduction and perceived behavioral control, with the latter association being more significant. Additionally, differences in salt reduction intention were observed among groups with different demographic characteristics, with higher mean levels of intention observed among hypertension patients with a family history of hypertension who lived in urban or rural suburban areas.

### 4.1 Differences in demographic characteristics

The present study found that, the intention to reduce salt intake was higher among middle-aged and older hypertensive patients aged 45 years or older in Chongqing (70.8%) than the proportion who were “not interested” in salt reduction in an international study involving seven countries (34%) (15), but significantly lower than in a survey based on the results of the China National Healthy Lifestyle Action WeChat Survey (90.2%) (16). This difference may be partly due to variations in the methodologies and target populations of the studies, and it highlights the significant potential for increasing the willingness of specific at-risk populations to reduce their salt intake. Complex cultural and cognitive mechanisms underlie differences in salt-reduction diets according to gender, family history of hypertension, and hometown. Etc. Specifically, men had lower perceived behavioral control over salt reduction than women, which may be related to women’s greater sense of control and self-efficacy over the amount of salt used when cooking for the family (17–19). Patients with a family history of hypertension had significantly higher mean salt reduction intention scores than those without, which may be attributed to their perceived risk of disease and stronger health beliefs (20). Additionally, salt reduction intentions were significantly higher among patients living in Central City, Chongqing Main City New Area, and Northeast Chongqing Three Gorges Reservoir, compared to those living in the towns in Southeast Chongqing Wuling Mountains Town Cluster. This reflects not only the differences in the availability of health resources but also the role of regional dietary culture and the socioeconomic environment on individual health beliefs and behavioral choices (21).

### 4.2 Association between attitudes and salt-reduction intentions

The present study confirms that a positive attitude toward a reduced-salt diet is an important factor in determining the intention of middle-aged and older hypertensive patients to reduce their salt intake. The WHO Global Report on sodium reduction highlights that reducing sodium intake is one of the most cost-effective ways to improve health and reduce the burden of non-communicable diseases, as it can prevent a significant number of cardiovascular events and deaths at a minimal total cost. The results of the present study show that more positive attitudes toward salt-reduction diets are associated with stronger intentions to follow such diets, which is consistent with previous studies (22, 23). Although salt reduction is often perceived by patients as a “negative” behavior that compromises tastes, the health benefits (e.g., blood pressure control) can be recognized and translated into positive behavioral intentions once acknowledged. Most consumers were willing to purchase salt-reduced foods even in the absence of a salt-reduction goal, suggesting that they perceived positive health outcomes from salt reduction (24). Residents with high levels of salt-reduction dietary attitudes were more likely to take the initiative to reduce salt intake (25, 26). Individuals’ evaluations of whether they approve or disapprove of a certain behavior influence their attitudes toward it, which ultimately affect its implementation (27). For example, they could be given targeted salt-reduction education to help them understand the main source of sodium in their diet (about 76% of salt intake in China comes from home cooking) (28). This would enable them to develop a correct understanding of a salt-reduction diet, fully grasp the harm caused by a high-salt diet, and change their negative attitudes toward it. If they already have a positive attitude toward a low-salt diet, the sodium-reduction education could further strengthen this.

### 4.3 Association between subjective norms and salt-reduction intentions

Unexpectedly, the subjective norms of a salt-reduced diet were not found to be significantly and directly associated with the intention to reduce salt intake in the present study. This is consistent with the findings of Alshagrawi et al. (29) in the field of vaccination, which suggest the existence of boundary conditions for the effect of social pressure. The unique communal eating culture and highly consistent “heavy” food preferences in Chongqing make it more difficult to obtain differentiated low-salt dietary advice or support from significant others (e.g., family and friends) (30, 31), which may diminish the predictive power of subjective norms. Furthermore, one study suggests that family disapproval acts as a deterrent to reducing salt intake (32, 33). However, this does not dismiss the role of social influence. A national research study found that exhortations from physicians and reminders from family members helped residents to begin reducing their salt intake (34). Therefore, future interventions may consider innovative strategies, such as integrating salt reduction advocacy into family or community group activities and establishing effective, positive social norms based on the local culture and environment.

### 4.4 Association between perceived behavioral control and salt-reduction intentions

The most critical finding of this study is that, in the theoretical model, perceived behavioral control is the strongest predictor of salt reduction dietary intentions in older hypertensive patients, with predictive power that exceeds that of attitudes. This aligns with the TPB expectation that, when behaviors are perceived as difficult to perform, an individual’s confidence in their own ability becomes the dominant factor in decision-making. Chongqing is one of the‘heavy’food cities and has a strong culture of gathering and eating. For middle-aged and older patients with hypertension, reducing salt intake involves overcoming multiple barriers, such as long-term taste dependence (35, 36) and the practical difficulties of obtaining low-sodium alternatives (37), as well as coping with “invisible salt” in pre-processed foods and when eating out (38). Therefore, high levels of PBC imply that patients believe they can overcome these challenges and are more likely to develop a firm intention to reduce their salt intake.

This finding has a central implication for interventions, namely that the most effective ones need to go beyond disseminating knowledge alone, which will not solve the real problem of a “sense of control.” Future interventions must systematically lower the behavioral threshold. Examples include promoting affordable low-sodium salt, encouraging restaurants to offer low-salt options, and teaching food label reading and low-salt cooking skills through community education programs. A targeted health communication campaign that taught these skills successfully encouraged 85% of respondents to report positive changes in their attitudes and behaviors, and 90% of participants developed a clear intention to reduce their salt intake (39). This strongly supports the feasibility of bridging the “intention-behavior gap” by enhancing PBC, given the strong association between behavioral control and desire-intention. Therefore, a multi-level empowerment system integrating policy support, environmental modification, and skill training for middle-aged and older hypertensive patients in Chongqing may be useful to transform salt reduction intentions into actual actions.

This cross-sectional study surveyed the dietary intention of salt reduction among hypertensive patients aged 45 and over in Chongqing City. The study used a self-constructed scale and had the following limitations. First, the cross-sectional design did not allow time-series relationships among the variables to be identified, making it difficult to infer causality. Secondly, although SEM analyses were able to test the hypothesized paths, the results were highly dependent on the quality of the measurements and the model setup and did not confirm causality; notably, the RMSEA value (0.083) was slightly above the ideal threshold, which may relate to sample size or model complexity, though other key indices (e.g., CFI, TLI) were acceptable and the core findings remain valid. Thirdly, the survey instrument was primarily based on the theoretical framework of planned behavior and existing literature. Qualitative interviews were not conducted in the prior period, nor was the Delphi expert consultation method refined, which may affect the scale’s measurement accuracy. Fourthly, potential selection bias due to convenience sampling, the data were derived from participants’ self-reports, which may have introduced information bias. Finally, due to geographical and condition-related limitations, the researchers were unable to conduct follow-up visits to assess patients’ actual behavioral changes after they had expressed their intention to reduce salt intake. Therefore, we recommend that future studies conduct reinterviews and expert consultations during the instrument development phase and use longitudinal designs wherever possible to track the dynamic relationship between intention and behavior.

## 5 Conclusion

In summary, this study validated a well-fitting model of dietary intention to reduce salt intake among middle-aged and older patients with hypertension in Chongqing, based on the theory of planned behavior. The study revealed that patients’ intention to reduce salt intake was moderated by family history of hypertension and urban–rural regional factors, highlighting the need for precise, population-based interventions. Additionally, perceived behavioral control emerged as the most critical psychosocial factor influencing salt reduction intention, surpassing the influence of attitude. However, subjective norms were not found to have a direct significant effect in the cultural context. This deepens the contextualized understanding of TPB in the field of dietary health and clarifies the priority targets for intervention. Therefore, future efforts to promote salt reduction should focus on empowering patients by systematically enhancing their sense of control through the creation of supportive environments and the upgrading of personal skills. It is also important to actively explore effective ways to construct positive social norms within local dietary cultures in order to promote the practical transformation of health intentions into enduring behaviors.

## Data Availability

The original contributions presented in the study are included in the article/Supplementary material, further inquiries can be directed to the corresponding author.
