# Supplementary material for: Intention to reduce dietary salt and its influencing factors in middle-aged and older hypertensive patients: a theory of planned behavior-based cross-sectional study
Source: Front Public Health. 2026 Mar 3;14:1765900. doi: 10.3389/fpubh.2026.1765900 (PMC12992000; doi:10.3389/fpubh.2026.1765900)
Supplement: Supplementary file 1 [file Table_1.doc]

**Research Questionnaire on Willingness and Influencing Factors of Salt-Reduction Dietary Behaviors Among Middle-Aged and Elderly (45+) Hypertensive Patients Based on the Theory of Planned Behavior**

Dear Uncle/Grandfather/Aunt/Grandmother:

Greetings! This is a research questionnaire conducted by the School of Public Health at Chongqing Medical University. The study focuses on salt-reduction dietary behaviors and their influencing factors among hypertensive patients aged 45 and above. Your responses will provide valuable data and insights for research on salt-reduction awareness and behaviors in hypertension management. Please read each question carefully and answer truthfully.

This study is ****anonymous****, and there are no right or wrong answers. Your honest opinions are greatly appreciated.

****Informed Consent Statement****
I have read this informed consent form and ****_________**** to participate in this survey.
○ Agree to participate
○ Do not agree to participate (skip to the end of the questionnaire)

***I. Basic Information***

Filling Instructions: Please check "√" next to the appropriate number based on your actual circumstances and enter the relevant information in the "______" field. Ensure no omissions occur during completion.

**1. Your year of birth: ______**

**2. Gender:** ①Male ②Female

**3. Your ethnicity:** ①Han Chinese ②Other ethnic group______

**4. What is your highest level of education?**

①Elementary school or below

②Junior high school

③High school or technical secondary school

④College (associate degree) or above

**5. What is your marital status?**

①Married

②Divorced

③Unmarried

③Widowed

**6. Where is your household residence located?**

①Rural

②Urban

1. **Who currently take care of your daily meals?**

①Self

②Spouse

③Children and/or in-laws

④Other relatives

⑤Housemaid/caregiver

⑥Others

1. **What is your current occupation? (If retired, please indicate your previous industry/employer)**

①Healthcare institution

②Education-related industry

③Commerce/Service sector

④Industry/manufacturing

⑤Government agencies and public institutions (excluding medical institutions and educational institutions)

⑥Agriculture,Forestry,animal husbandry, and fisheries

⑦Other (please specify) ______

**9. What is your monthly income? (Including salary, pensions, social security, family support, and government subsidies)**

① <1,000 yuan

② 1,000–3,000 yuan

③ 3,001–5,000 yuan

④ 5,001–8,000 yuan

⑤ >8,000 yuan

**10. Your height is ______ (unit: cm), and your most recent measured weight is ______ (unit: kg)(Please retain one decimal place, e.g., height 171.5 cm, weight 65.5 kg)**

**11. How many years have passed since you were first diagnosed with hypertension? ______ year.How many years have you been taking antihypertensive medication?______ year.**

**12. Do you have a family history of hypertension?**

① Yes

② No

**13. In addition to hypertension, do you have any of the following conditions?**

① Hyperlipidemia

② Diabetes

③ Coronary heart disease

④ Stroke

⑤ Other conditions

⑥ None

**14. Are you currently taking traditional Chinese medicine (TCM) or Western medicine for blood pressure control?**

① Traditional Chinese medicine

② Western medicine

③ Both TCM and Western medicine

**15. How frequently do you smoke?**

① Never smoke

② More than 5 cigarettes per day

③ 1–5 cigarettes per day

④ More than 1 cigarette per week but less than 1 per day

⑤ Less than 1 cigarette per week

⑥ Already quit smoking

**16. How often do you drink alcohol?**

① Every day

② 3–6 days/week

③ 1–2 days/week

④ 1–3 days/month

⑤ Less than 1 day/month

⑥ Never drink alcohol

⑦ Have quit drinking

**17. Have you ever promoted low-salt diet to people around you?**

① Never

② Rarely

③ Occasionally

④ Frequently

**18. How often do you use low-sodium salt?**

① Never

② Rarely

③ Occasionally

④ Frequently

**19. When purchasing food, how often do you check the salt/sodium content on nutrition labels?**

① Never

② Rarely

③ Occasionally

④ Frequently

**20. How often do you use a measured salt spoon?**

① Never

② Rarely

③ Occasionally

④ Frequently

***II. Theory of Planned Behavior (TPB) Scale***

For each statement below, the numbers represent increasing levels of agreement, where **"1" = Strongly Disagree** and **"5" = Strongly Agree**. Higher scores indicate stronger agreement. The meaning of each extreme value is clearly labeled:

| 1 | 2 | 3 | 4 | 5 |
| --- | --- | --- | --- | --- |
| Strongly disagree | Disagree | neutral | agree | Strongly agree |

Please read each statement carefully and mark "✓" or circle "〇" on the number that best represents your actual situation.

| Dimension | Item | Strongly disagree | Disagree | neutral | agree | Strongly agree |
| --- | --- | --- | --- | --- | --- | --- |
| Salt Reduction Dietary Intentions | INT1.I am very concerned about the impact of high-salt diets on blood pressure. |  |  |  |  |  |
|  | INT2.I intend to start a low-salt diet as soon as possible. |  |  |  |  |  |
|  | INT3.I hope to maintain a low-salt diet for more than four weeks. |  |  |  |  |  |
| perceived behavioral control of salt reduction | PBC1.I am confident that I can maintain a low-salt diet over the next 4 weeks. |  |  |  |  |  |
|  | PBC2.Whether I maintain a low-salt diet in the next four weeks depends on whether I can control my own behavior. |  |  |  |  |  |
|  | PBC3.Over the next four weeks, it will be difficult for me to follow a low-salt diet. |  |  |  |  |  |
| attitude towards salt reduction | ATT1. If I maintain a low-salt diet, I can reduce my risk of stroke. |  |  |  |  |  |
|  | ATT2.If I maintain a low-salt diet, I can reduce my chances of developing heart disease. |  |  |  |  |  |
|  | ATT3.If I maintain a low-salt diet, my overall health will improve. |  |  |  |  |  |
|  | ATT4.In my opinion, a low-salt diet represents healthier eating habit. |  |  |  |  |  |
| subjective norms of salt reduction | SN1. My family members advise me to maintain a low-salt diet. |  |  |  |  |  |
|  | SN2.My friends with hypertension recommend that I maintain a low-salt diet |  |  |  |  |  |
|  | SN3.My primary doctor for hypertension advises me to maintain a low-salt diet. |  |  |  |  |  |
